# A survey of Italian cat owners’ attitudes towards cat vaccination through a web-based questionnaire

**DOI:** 10.1186/s12917-021-02981-z

**Published:** 2021-08-09

**Authors:** Joel Fernando Soares Filipe, Stefania Lauzi, Lucrezia Pina, Paola Dall’Ara

**Affiliations:** Department of Veterinary Medicine, University of Milan, Via dell’Università 6, 26900 Lodi, Italy

**Keywords:** Vaccination, Cat, Prevention, Infectious diseases, Questionnaire

## Abstract

**Background:**

Vaccination plays an important role in feline healthcare as it is the most effective measure for prevention against feline infectious diseases. Therefore, it is important to know owners’ opinion towards cats’ vaccination and current veterinary practices in order to advice owners on the use of the correct vaccination protocol.

This study aimed to investigate the proportion of cats regularly vaccinated and identify the main factors motivating cat owners’ decisions related to vaccination in Italy. A questionnaire was disseminated online (mainly via social networks) to collect data regarding Italian cat owners’ demographics, information about cats, factors regarding cats’ vaccination, and veterinary-owner relationship.

**Results:**

A total of 1264 owners participated in the survey and 1247 questionnaires were completed and statistically analyzed. The majority (91%; *n* = 1131) of cats were vaccinated and 80% (*n* = 998) had been vaccinated within the last 3 years. Age of 2–4 years old cats and the acquisition from a breeder or cat shop were significantly associated with vaccination within the last 3 years. Cats vaccinated but not within the last 3 years were significantly associated with cat’s indoor lifestyle, cats’ age ≥ 5 years old and low annual household income. Importance of vaccination cost, low annual household income and owners’ job not related to healthcare was statistically associated with the lack of cat’s vaccination. In addition, 86% of the owners took their cat regularly to veterinary clinics. Veterinarians play a significant role in owners’ decision, and they are considered the most useful source of information about vaccination by 97% of owners.

**Conclusions:**

The high number of recently vaccinated cats suggests owner’s attention towards feline vaccination and cat’s health. The importance of veterinarian’s advice along with the knowledge of factors associated to the unvaccinated status of cats may help veterinarians to grow owner’s confidence and increase prevention of feline infectious diseases.

However further investigations based on a more comprehensive sample of the general population are needed to confirm the results of this survey.

**Supplementary Information:**

The online version contains supplementary material available at 10.1186/s12917-021-02981-z.

## Background

Vaccination is the most important measure for infectious diseases’ prevention and has become an integral part of pet healthcare. According to the international vaccination guidelines drawn up and shared by various pet veterinary association (e.g., World Small Animal Veterinary Association, − WSAVA, American Association of Feline Practitioners – AAFP, Advisory Board on Cat Diseases - ABCD), all cats, regardless of circumstances or geographical location, should be vaccinated against feline parvovirus (FPV), feline calicivirus (FCV) and feline herpesvirus-1 (FHV-1) [1–4]. These vaccines have been defined “core vaccines”. In areas of the world where rabies virus infection is endemic, vaccination against this pathogen should also be considered core. For those cats living in certain geographic locations, local environment, or lifestyle at risk of specific infections, “non-core vaccines” should be also given. The “non-core vaccines” are the vaccines against feline leukemia virus (FeLV), *Chlamydophila* (*Chlamydia) felis*, *B. bronchiseptica* and feline immunodeficiency virus (FIV - the last two vaccines are not available in Italy). The non-core vaccination against FeLV can be considered “circumstantial” and promoted to core based on geographical situation of different countries and suggested for all kittens up to and including 1 year of age, and for at-risk adult cats (e.g. cats with outdoor access, living with other cats or regularly visiting a boarding cattery). The vaccine against feline infectious peritonitis (FIP) is defined as “not recommended” [1–4]. In Italy, vaccinations against FPV, FCV and FHV-1 are “core” and periodically administered to all kittens and cats, while FeLV vaccination is considered circumstantial (that is core) and is recommended for kittens and all adult cats at risk. Finally, rabies vaccination in Italy is mandatory only for travels abroad.

Number of pets is increasing in Italy and the most recent reports estimate almost 7.3 million cats (1 cat every 3.5 families) in our country, and 50.3% of Italians have at least one cat [5]. The routine vaccination programs in Italy have led to a decline in the frequency and severity of some feline infectious diseases and have reduced feline morbidity and mortality [6]. Compliance of cat owners with vaccination is also an important factor leading to vaccination programs following international guidelines [7].

However, pet owners’ concerns regarding vaccination and prevention have increased in Italy in the past 10 years [8, 9]. In human medicine, an anti-vaccination movement has been observed [10]. The fall-off in vaccinations has been linked to recent measles outbreaks [11] also in Italy [12], and the World Health Organization (WHO) has named vaccine hesitancy as one of the top 10 global health threats. It has been suggested that pet owners may have similar concerns regarding vaccination of cats and dogs, and movements discouraging vaccination of pets have been recently reported [13–15].

It is important to evaluate up-to-date owners’ attitude towards vaccination of cats. Cat owners’ vaccination compliance in Northern Europe has been previously evaluated in two studies, one from the United Kingdom (UK) [16], and the other from Germany [7]. To date, studies on vaccination compliance of Italian pet owners, in a country where the non-vaccination movement has been well reported in human medicine [12], have not been performed. Therefore, the aim of this study was to evaluate the proportion of vaccinated cats in Italy and understand the factors influencing owners’ decisions related to vaccination of cats in Italy through a web-based questionnaire, with special emphasis on the importance of the relationship between owner and veterinarian.

## Results

### Participation in the survey

A total of 1264 questionnaires were collected. Not all participants answered all questions, and consequently not all participants were included in the study. Answers of owners younger than 16 years of age (*n* = 3) and owners of cats under 8 weeks of age (*n* = 2) were excluded. Moreover, answers of respondents who had not answered to the question about the vaccination status of the cats (*n* = 10) and to the question about the last vaccination (*n* = 2) were also excluded as this information was essential for data analysis.

At the end of this first control, a total of 1247 out of 1264 questionnaires were statistically analyzed.

### Description of cats’ owners

The majority of cats’ owners were females (92%; *n* = 1145/1247), 21–49 years old (75%; *n* = 941/1247) and from Northern Italy (73%; *n* = 810/1247). More than half of the owners (65%; *n* = 805/1236) belonged to a family with a high level of education (bachelor’s or Master/post-university degree).

The cats of the majority of respondents (91%; *n* = 1131/1247) had been vaccinated at least once in their lifetime, and 80% (*n* = 998/1247) of owners had their cats vaccinated within the last 3 years (2015–2018).

The demographic characteristics of the owners according to their decision regarding the vaccination status of their cats are summarized in Table 1.

**Table 1:** Description of the cats’ owners, including univariate analysis *p*-values for association with the cat’s vaccination status

Question	Response option	Total (%)	No. (%) recently vaccinated (in the last 3 years)	No. (%) not recently vaccinated (over the last 3 years)	No. (%) unvaccinated	*P*-value**
**Age of respondent**	17–20 years	22 (2)	12 (1)	4 (3)	6 (5)	***0.083***
	21–29 years	302 (24)	240 (24)	30 (23)	32 (28)	
	30–39 years	328 (26)	257 (26)	45 (34)	26 (22)	
	40–49 years	311 (25)	254 (25)	27 (20)	30 (26)	
	50–59 years	204 (16)	165 (17)	19 (14)	20 (17)	
	≥ 60 years	80 (7)	70 (7)	8 (6)	2 (2)	
	*Total*	*1247*	*998*	*133*	*116*	
**Gender**	Female	1145 (92)	915 (92)	122 (92)	108 (94)	0.736
	Male	98 (8)	80 (8)	11 (8)	7 (6)	
	*Total*	*1243*	*995*	*133*	*115*	
**Living area of respondent based on number of inhabitants**	City (≥15,000 people)	835 (63)	605 (61)	74 (56)	69 (60)	0.548
	Town (< 15,000 people)	498 (37)	393 (39)	59 (44)	46 (40)	
	*Total*	*1333*	*998*	*133*	*115*	
**Living area of respondent based on postal code**	North Italy	810 (73)	639 (71)	98 (82)	73 (74)	0.701
	Middle Italy	108 (10)	97 (11)	5 (4)	6 (6)	
	South Italy	106 (9)	90 (10)	7 (6)	9 (9)	
	Abroad^a^	91 (8)	70 (8)	10 (8)	11 (11)	
	*Total*	*1115*	*896*	*120*	*99*	
**Level of education of respondent**	Primary/Middle/High school certificate	671 (54)	526 (53)	80 (60)	65 (56)	***0.197***
	Bachelor’s degree	470 (38)	387 (39)	38 (29)	45 (39)	
	Master/Post-university degree	103 (8)	82 (8)	15 (11)	6 (5)	
	*Total*	*1244*	*995*	*133*	*116*	
**Highest level of education in the household of respondent**	Primary/Middle/High school certificate	431 (35)	332 (34)	45 (34)	54 (47)	0.213
	Bachelor’s degree	588 (47)	479 (48)	60 (45)	49 (42)	
	Master/Post-university degree	217 (18)	177 (18)	27 (21)	13 (11)	
	*Total*	*1236*	*988*	*132*	*116*	
**Job of respondent**	Healthcare job	232 (22)	190 (22)	30 (26)	12 (13)	***0.004***
	Student	191 (18)	145 (17)	27 (24)	19 (20)	
	Other	650 (60)	529 (61)	57 (50)	64 (67)	
	*Total*	*1073*	*998*	*133*	*116*	
**Annual household income of respondent**	≤ 9000 €	76 (7)	53 (6)	9 (7)	14 (13)	***< 0.001***
	10–29,000 €	546 (49)	420 (47)	67 (55)	59 (57)	
	30–49,000 €	339 (30)	285 (32)	33 (27)	21 (20)	
	≥ 50,000 €	156 (14)	133 (15)	13 (11)	10 (10)	
	*Total*	*1117*	*891*	*122*	*104*	
**Number of children (≤ 13 years old) in the household of respondent**	None	1009 (82)	806 (82)	112 (85)	91 (82)	***0.143***
	≥ 1	223 (18)	180 (18)	19 (15)	24 (18)	
	*Total*	*1232*	*986*	*131*	*115*	
**How were you informed about the questionnaire?**	Internet (social networks included)	1031 (85)	833 (85)	107 (82)	91 (80)	0.520
	Relatives/friends	168 (14)	127 (13)	20 (15)	21 (18)	
	Others	7 (1)	1 (2)	4 (3)	2 (2)	
	*Total*	*1206*	*983*	*131*	*114*	

** significant results and tendency are reported in bold

^a^ abroad = Italian owners living in another country with their cat

### Description of cats

The majority of cat’s owners possessed more than one cat (69%; *n* = 854/1240) and had acquired it as a kitten (91%; *n* = 1129/1235). Most of the cats were previously stray cats or cats from shelters (57%; *n* = 703/1239), whereas few cats (18%; *n* = 225/1239) came from a breeder or a pet shop. Only 23% (*n* = 278/1233) were purebred cats. The majority of cats lived indoor (63%; *n* = 787/1240) or had a mixed lifestyle indoor with outdoor access (35%; *n* = 430/1240). One third of cats (34%; *n* = 424/1239) had traveled or had attended a cat show or a cattery.

A summary of the cats’ characteristics according to their vaccination status is summarized in Table 2.

**Table 2:** Cats’ description, including univariate analysis *p*-values for association with the cat’s vaccination status

Question	Response option	Total (%)	No. (%) recently vaccinated (in the last 3 years)	No. (%) not recently vaccinated (over the last 3 years)	No. (%) unvaccinated	*P*-value*
**Number of cats owned by respondent**	1	386 (31)	296 (30)	43 (32)	47 (41)	***0.068***
	2	377 (31)	314 (32)	38 (29)	25 (21)	
	3	187 (15)	142 (14)	23 (17)	22 (19)	
	≥ 4	290 (23)	239 (24)	29 (22)	22 (19)	
	*Total*	1240	991	133	116	
**Age of the cat**	8 weeks – 1 year	196 (16)	171 (17)	1 (1)	24 (21)	***< 0.001***
	2–4 years	485 (39)	445 (45)	7(5)	33 (28)	
	5–9 years	371 (30)	264 (27)	71 (53)	36 (31)	
	≥ 10 years	184 (15)	107 (11)	54 (41)	23 (20)	
	*Total*	*1236*	*987*	*133*	*116*	
**Age of the cat at acquisition**	≤1 year	1129 (91)	910 (92)	120 (90)	99 (85)	0.558
	2–4 years	69 (6)	51 (5)	9 (7)	9 (8)	
	≥ 5 years	37 (3)	25 (3)	4 (3)	8 (7)	
	*Total*	*1235*	*986*	*133*	*116*	
**Origin of the cat**	Breeder/shop	225 (18)	215 (22)	6 (5)	4 (3)	***0.001***
	Animal shelter/charity/stray	703 (57)	534 (54)	93 (70)	76 (66)	
	Relatives/friends	260 (21)	200 (20)	29 (22)	31 (27)	
	Internet	50 (4)	42 (4)	4 (3)	4 (4)	
	*Total*	*1238*	*991*	*132*	*115*	
**Purebred cat**	No	955 (77)	727 (74)	120 (90)	108 (93)	0.204
	Yes	278 (23)	257 (26)	13 (10)	8 (7)	
	*Total*	*1233*	*984*	*133*	*116*	
**Indoor/outdoor access**	Indoor only	787 (63)	648 (65)	77 (58)	62 (53)	***0.017***
	Indoor and outdoor	430 (35)	330 (34)	52 (39)	48 (42)	
	Outdoor only	23 (2)	13 (1)	4 (3)	6 (5)	
	*Total*	*1240*	*991*	*133*	*116*	
**Previous cat’s experience**	Cattery/cat show	182 (15)	168 (17)	12 (9)	2 (2)	***< 0.001***
	Travel	242 (19)	199 (20)	28 (21)	15 (13)	
	None	815 (66)	623 (63)	93 (70)	99 (85)	
	*Total*	*1239*	*990*	*133*	*116*	
**Future cat’s experience**	Cattery/cat show	164 (13)	156 (16)	1 (1)	7 (6)	0.232
	Travel	233 (19)	202 (21)	20 (15)	11 (9)	
	None	831 (68)	622 (63)	111 (84)	98 (85)	
	*Total*	*1228*	*980*	*132*	*116*	

* significant results and tendency are reported in bold

### Owners’ attitudes towards vaccination

Overall, respondents identified as “important” or “very important” factors for vaccination the veterinary advice (87%; *n* = 1021/1179), infectious disease susceptibility (88%; *n* = 1022/1157), and vaccination efficacy (89%; *n* = 1032/1159), while the time to bring the cat to the veterinary clinic was considered “unimportant” (91%; *n* = 1053/1160).

In this survey, owners were asked not only if their cat had been vaccinated but also how long ago they had been vaccinated, according to the vaccination frequency suggested by the international guidelines (WSAVA, AAFP, ABCD) for the core vaccines [1, 2, 4]. Cats that had been vaccinated in 2015–2018 were classified as “recently vaccinated”, whereas cats that had been vaccinated before 2015 were classified as “not recently vaccinated”.

The main factors related to owners’ decision of vaccinate or not their cats are reported in Table 3.

**Table 3:** Importance of factors influencing the decision on vaccination of cats among cat owners, including univariate analysis p-values for association with the cat’s vaccination status

Factor	Response option^a^	Total (%)	No. (%) recently vaccinated (in the last 3 years)	No. (%) not recently vaccinated (over the last 3 years)	No. (%) unvaccinated	*P*-value**
**Cost**	Unimportant	1036 (86)	862 (89)	99 (77)	75 (69)	***< 0.001***
	Important	167 (14)	104 (11)	29 (23)	34 (31)	
	*Total*	*1203*	*966*	*128*	*109*	
**Possible side effects**	Unimportant	381 (32)	312 (33)	34 (27)	35 (35)	0.333
	Important	798 (68)	639 (67)	93 (73)	66 (65)	
	*Total*	*1179*	*951*	*127*	*101*	
**Cat’s stress**	Unimportant	561 (48)	474 (50)	44 (35)	43 (48)	0.216
	Important	605 (52)	464 (50)	82 (65)	59 (52)	
	*Total*	*1166*	*938*	*126*	*102*	
**Veterinarian’s advice**	Unimportant	158 (13)	120 (13)	17 (14)	21 (20)	0.225
	Important	1021 (87)	830 (87)	108 (86)	83 (80)	
	*Total*	*1179*	*950*	*125*	*104*	
**Cat’s susceptibility to infectious diseases**	Unimportant	135 (12)	103 (11)	14 (11)	18 (18)	***0.150***
	Important	1022 (88)	830 (89)	110 (89)	82 (82)	
	*Total*	*1157*	*933*	*124*	*100*	
**Infectious diseases’ level of risk**	Unimportant	96 (8)	76 (8)	9 (7)	11 (11)	0.594
	Important	1065 (92)	860 (92)	115 (93)	90 (89)	
	*Total*	*1161*	*936*	*124*	*101*	
**Efficacy of vaccination**	Unimportant	127 (11)	92 (10)	16 (13)	19 (20)	0.220
	Important	1032 (89)	844 (90)	110 (87)	78 (80)	
	*Total*	*1159*	*936*	*126*	*97*	
**Time to take the cat to the veterinary clinic**	Unimportant	1053 (91)	873 (9)	104 (82)	76 (77)	***< 0.001***
	Important	107 (9)	62 (7)	22 (18)	23 (23)	
	*Total*	*1160*	*935*	*126*	*99*	
**Cat’s lifestyle**	Unimportant	482 (41)	418 (44)	25 (20)	39 (38)	***< 0.001***
	Important	690 (59)	524 (56)	103 (80)	63 (62)	
	*Total*	*1172*	*942*	*128*	*102*	
**Cat’s age**	Unimportant	515 (44)	431 (46)	32 (25)	52 (50)	0.242
	Important	655 (56)	508 (54)	95 (75)	52 (50)	
	*Total*	*1170*	*939*	*127*	*104*	
**Current cat’s disease/therapy**	Unimportant	212 (18)	158 (17)	21 (17)	33 (33)	0.355
	Important	954 (82)	781 (83)	106 (83)	67 (67)	
	*Total*	*1166*	*939*	*127*	*100*	

** significant results and tendency are reported in bold

^a^ The word “Unimportant” combines the answers “not important” and “not very important” - The word “Important” combines the answers “important” and “very important”

### Owner-veterinarian relationship and veterinary recommendations

The majority of respondents (86%; *n* = 1041/1213) took often their cat to a veterinarian (at least once a year), and veterinarians were considered the most important source of information about the cat’s health by 97% (*n* = 1194/1227) of them (overcoming books, 86%; *n* = 960/1114, and internet, 68%; *n* = 811/1145).

Few cats’ owners decided not to take their cats to a veterinarian (14%; *n* = 172/1213) and the main reason was the time involved with a visit to the veterinarian and the long distance from the veterinary clinics (53%; *n* = 91/172).

Depending on the veterinarians, vaccination against at least one disease is suggested every year (59%; *n* = 728/1238), every 2 years (13%; *n* = 162/1238) or every 3 years (13%; *n* = 156/1238).

Most owners (62%; *n* = 770/1242) were not aware of the possibility to evaluate the duration of immunity (DOI), however most of them (84%; *n* = 1031/1233) were further in agreement with the possible use of a DOI test in the future.

Results of the relationship between owners and veterinarians according to the vaccination status of cats are presented in Table 4.

**Table 4:** Owner-veterinarian relationship and veterinary recommendations, including univariate analysis p-values for association with the cat’s vaccination status

Question	Response option^a^		Total (%)	No. (%) recently vaccinated (in the last 3 years)	No. (%) not recently vaccinated (over the last 3 years)	No. (%) unvaccinated	*P*-value**
**Revaccination recommendation by the veterinarian**	Every year		728 (59)	650 (65)	43 (33)	35 (31)	***< 0.001***
	Every two/three years		321 (26)	280 (28)	34 (26)	7 (6)	
	More than three years		20 (1)	8 (1)	8 (6)	2 (2)	
	Unknown		171 (14)	57 (6)	46 (35)	68 (61)	
	*Total*		*1240*	*995*	*131*	*112*	
**Main factors for not making an appointment with the veterinarian**	Cost		12 (1)	6 (1)	3 (2)	3 (3)	0.207
	The cat has never had health problems		60 (5)	31 (3)	19 (14)	10 (9)	
	Finding and catching the cat/cat’s stress		9 (1)	1 (0)	5 (4)	3 (3)	
	Distance to clinic and transport, waiting time, opening hours		91 (7)	58 (6)	16 (12)	17 (16)	
	None (I go to the veterinarian)		1041 (86)	877 (90)	89 (68)	75 (69)	
	*Total*		*1213*	*973*	*132*	*108*	
**Value of source of information about vaccination**	Internet	Useless	334 (32)	269 (29)	37 (29)	28 (26)	0.804
		Useful	712 (68)	545 (67)	89 (71)	78 (74)	
		*Total*	*1046*	*814*	*126*	*106*	
	Books	Useless	154 (14)	119 (13)	17 (14)	18 (18)	0.887
		Useful	960 (86)	775 (87)	104 (86)	81 (82)	
		*Total*	*1114*	*894*	*121*	*99*	
	Relatives/friends	Useless	654 (60)	525 (60)	75 (64)	54 (54)	0.621
		Useful	441 (40)	351 (40)	43 (36)	47 (46)	
		*Total*	*1095*	*876*	*118*	*101*	
	Breeder	Useless	431 (40)	338 (39)	61 (51)	32 (32)	0.664
		Useful	658 (60)	533 (61)	58 (49)	67 (68)	
		*Total*	*1089*	*871*	*119*	*99*	
	Veterinarian	Useless	33 (3)	24 (2)	5 (4)	4 (4)	0.337
		Useful	1203 (97)	959 (98)	125 (96)	110 (96)	
		*Total*	*1236*	*983*	*130*	*114*	
	Pet shop	Useless	677 (63)	555 (65)	80 (68)	42 (41)	0.679
		Useful	404 (37)	306 (35)	38 (32)	60 (59)	
		*Total*	*1081*	*861*	*118*	*102*	
	Animal shelter/charity	Useless	301 (27)	228 (26)	44 (39)	29 (28)	0.421
		Useful	799 (73)	656 (74)	68 (61)	75 (72)	
		*Total*	*1100*	*884*	*112*	*104*	
	Family doctor	Useless	837 (78)	668 (78)	100 (86)	69 (68)	0.203
		Useful	238 (22)	190 (22)	16 (14)	32 (32)	
		*Total*	*1075*	*858*	*116*	*101*	
	Pharmacist	Useless	765 (71)	617 (72)	89 (76)	59 (58)	0.427
		Useful	312 (29)	242 (28)	28 (24)	42 (42)	
		*Total*	*1077*	*859*	*117*	*101*	
**Duration of Immunity (DOI) test knowledge**	Yes		472 (38)	385 (39)	57 (43)	30 (26)	***0.010***
	No		770 (62)	608 (61)	76 (57)	86 (74)	
	*Total*		1242	993	133	116	
**Possible use of DOI test**	Yes		1031 (84)	840 (85)	108 (81)	83 (84)	***0.004***
	No		202 (16)	146 (15)	25 (19)	31 (16)	
	*Total*		*1233*	*986*	*133*	*114*	

** Significant results and tendency are reported in bold

^a^ The word “Useful” combines the answers “useful” and “very useful”

### Factors with positive and negative association with the vaccination status of cats

Univariable regression was used to test the independent variables for association with cat’s vaccination status. Variables that were correlated or that did not improve the fit of the model (e.g. age of cat at acquisition, postal code of the respondent, highest level of education in the household) were excluded. The association of 17 factors with the vaccination status of cats was evaluated in a multinomial logistic regression. Results of the statistical analysis (*p*-values and OR) are reported in Tables 5 and 6. The adult age of the cat (2–4 years), the origin from either a breeder or a pet shop, the low importance for vaccination cost and a high annual household income had the highest and the most significant positive association on the recently vaccinated status. Participation to a cat show and higher compliance with possible use of DOI tests tended to be factors associated to recent vaccination (Table 5). Importance of cat’s lifestyle (indoor only versus outdoor lifestyle), 5–9 years of age and low annual household income were important factors associated with not recent vaccination of cats (Tables 5 and 6). Importance of vaccination cost, low annual household income, high percentage of owners working in jobs other than being a student or working in healthcare had the most significant impact on the unvaccinated status (Table 6). Owners aged 21–49 years tended to be more prone to not vaccinate their cats. The young age (8 weeks-1 year) and the age 5–9 years of cats tended to be associated to the unvaccinated status when compared with recently vaccinated cats. The lower percentage of owners with a high household income tended to be associated to the unvaccinated status compared to the not recently vaccinated cats (Tables 5 and 6).

**Table 5:** Statistical analysis (multinomial logistic regression) of the results obtained from the online questionnaire using the cats recently vaccinated as the reference category

Question	Response option^a^		Cat not recently vaccinated		Cat unvaccinated	
			*p-value*	OR (95% Cl)	*p-value*	OR (95% Cl)
**Origin of the cat**	Breeder/shop		**0.024**	0.239 (0.069–0.831)	0.764	0.788 (0.166–3.742)
	Animal Shelter/charity/stray		0.572	1.207 (0.627–2.324)	0.628	1.221 (0.545–2.731)
	Internet		0.836	0.819 (0.125–5.386)	0.602	1.536 (0.306–7.697)
	Relatives/friends			1		1
**Number of cats owned by respondent**	1		0.517	0.818 (0.445–1.503)	0.511	1.270 (0.623–2.588)
	≥ 2			1		1
**Age of the cat**	8 weeks – 1 year		**< 0.001**	0.008 (0.001–0.070)	0.090	0.397 (0.136–1.157)
	2–4 years		**< 0.001**	0.019 (0.006–0.059)	**< 0.001**	0.128 (0.044–0.377)
	5–9 years		**0.032**	0.509 (0.274–0.945)	0.055	0.389 (0.149–1.021)
	≥ 10 years			1		1
**Indoor/outdoor access**	Indoor only		0.441	1.269 (0.692–2.330)	0.138	0.570 (0.271–1.199)
	Indoor and outdoor			1		1
**Previous cat’s experience**	Cattery/cat shows		0.728	0.833 (0.297–2.333)	0.092	0.131 (0.012–1.396)
	Travel		0.543	0.807 (0.403–1.613)	0.284	0.599 (0.234–1.530)
	None			1		1
**Importance of factors influencing the vaccinated cats’ owners **	Cost	Important	0.234	1.591 (0.740–3.421)	**0.042**	2.266 (1.030–4.989)
		Unimportant		1		1
	Veterinarian’s advice	Important	0.230	0.497 (0.159–1.557)	0.128	0.366 (0.100–1.337)
		Unimportant		1		1
	Time necessary to vaccinate the cat	Important	0.167	1.858 (0.771–4.476)	0.124	2.161 (0.810–5.767)
		Unimportant		1		1
	Cat’s lifestyle	Important	**0.010**	2.801 (1.275–6.151)	0.242	1.703 (0.698–4.153)
		Unimportant		1		1
	Infectious diseases’ level of risk	Important	0.185	0.429 (0.123–1.501)	0.816	0.846 (0.208–3.447)
		Unimportant		1		1
**Duration of Immunity (DOI) test knowledge**	Yes		0.984	1.007 (0.506–2.004)	0.295	0.643 (0.282–1.469)
	No			1		1
**Possible use of DOI test**	Yes		0.083	0.535 (0.264–1.084)	0.226	0.595 (0.257–1.379)
	No			1		1
**Age of respondent**	17–20 years		0.936	1.084 (0.151–7.783)	0.244	0.340 (0.055–2.088)
	21–49 years		0.285	0.641 (0.284–1.448)	0.072	0.412 (0.157–1.081)
	≥ 50 years			1		1
**Level of education of respondent**	Primary/middle school certificate		0.392	0.278 (0.015–5.206)	0.739	0.604 (0.031–11.764)
	High school certificate		0.713	1.112 (0.632–1.958)	0.193	1.610 (0.786–3.296)
	Bachelor/Master/PhD			1		1
**Job of respondent**	Other		0.054	0.497 (0.244–1.013)	0.257	1.696 (0.681–4.225)
	Students		0.993	1.006 (0.291–3.477)	0.876	1.205 (0.116–12.500)
	Healthcare			1		1
**Annual household income of respondent**	≤ 9000 €		0.108	3.278 (0.771–13.932)	0.032	5.036 (1.152–22.022)
	10–29,000 €		**0.011**	3.605 (1.350–9.629)	0.121	2.448 (0.790–7.581)
	30–49,000 €		0.172	2.017 (0.736–5.529)	0.405	0.583 (0.164–2.078)
	≥ 50,000 €			1		1
**Children (≤ 13 years old) in the household of respondent**	Yes		0.711	0.857 (0.379–1.940)	0.693	1.186 (0.508–2.769)
	No			1		1

Significant results (*p* < 0.05) are reported in bold

*Cl* Confidence interval, *OR* Odds ratio

^a^The word “Unimportant” combines the answers “not important” and “not very important” - The word “Important” combines the answers “important” and “very important”

**Table 6:** Statistical analysis (multinomial logistic regression) of the results obtained from the online questionnaire using the unvaccinated cats as the reference category

Question	Response option^a^		Cat not recently vaccinated		Cat recently vaccinated	
			*p-value*	OR (Cl 95%)	*p-value*	OR (Cl 95%)
**Origin of the cat**	Breeder/shop		0.196	0.303 (0.050–1.850)	0.764	1.269 (0.267–6.025)
	Animal Shelter/charity/stray		0.982	0.989 (0.393–2.489)	0.628	0.819 (0.366–1.833)
	Internet		0.587	0.533 (0.055–5.149)	0.602	0.651 (0.130–3.263)
	Relatives/friends			1		1
**Number of cats owned by respondent**	1		0.302	0.644 (0.279–1.485)	0.511	0.788 (0.386–1.605)
	≥ 2			1		1
**Age of the cat**	8 weeks – 1 year		**0.001**	0.020 (0.002–0.198)	0.090	2.521 (0.865–7.349)
	2–4 years		**0.006**	0.151 (0.039–0.585)	**< 0.001**	7.789 (2.650–22.897)
	5–9 years		0.584	1.307 (0.500–3.416)	0.055	2.567 (0.980–6.727)
	≥ 10 years			1		1
**Indoor/outdoor access**	Indoor only		0.063	2.229 (0.956–5.194)	0.138	1.756 (0.834–3.697)
	Indoor and outdoor			1		1
**Previous cat’s experience**	Cattery/cat shows		0.133	6.376 (0.568–71.585)	0.092	7.656 (0.716–81.821)
	Travel		0.575	1.347 (0.476–3.811)	0.284	1.670 (0.654–4.265)
	None			1		1
**Importance of factors influencing the vaccinated cats’ owners**	Cost	Important	0.447	0.702 (0.282–1.748)	**0.042**	0.441 (0200–0.971)
		Unimportant		1		1
	Veterinarian’s advice	Important	0.666	1.357 (0.339–5.433)	0.128	2.729 (0.748–9.957)
		Unimportant		1		1
	Time necessary to vaccinate the cat	Important	0.781	0.860 (0.296–2.496)	0.124	0.463 (0.173–1.235)
		Unimportant		1		1
	Cat’s lifestyle	Important	0.362	1.645 (0.564–4.795)	0.242	0.587 (0.241–1.433)
		Unimportant		1		1
	Infectious diseases’ level of risk	Important	0.396	0.507 (0.106–2.433)	0.816	1.182 (0.290–4.813)
		Unimportant		1		1
**Duration Of Immunity (DOI) test knowledge**	Yes		0.348	1.566 (0.614–3.993)	0.295	1.554 (0.681–3.550)
	No			1		1
**Possible use of DOI test**	Yes		0.822	0.899 (0.356–2.271)	0.226	1.679 (0.725–3.890)
	No			1		1
**Age of respondent**	17–20 years		0.304	3.184 (0.351–28.925)	0.244	2.938 (0.479–18.029)
	21–49 years		0.424	1.557 (0.526–4.605)	0.072	2.429 (0.925–6.378)
	≥ 50 years			1		1
**Level of education of respondent**	Primary/middle school certificate		0.632	0.460 (0.019–10.966)	0.739	1.657 (0.085–32.292)
	High school certificate		0.377	0.691 (0.304–1.570)	0.193	0.621 (0.303–1.272)
	Bachelor/Master/PhD			1		1
**Job of respondent**	Other		**0.018**	0.293 (0.106–0.810)	0.257	0.590 (0.237–1.469)
	Student		0.883	0.834 (0.075–9.245)	0.876	0.830 (0.080–8.606)
	Healthcare			1		1
**Annual household income of respondent**	≤ 9000 €		0.628	0.651 (0.114–3.702)	**0.032**	0.199 (0.045–0.868)
	10–29,000 €		0.556	1.473 (0.406–5.345)	0.121	0.409 (0.132–1.265)
	30–49,000 €		0.094	3.459 (0.810–14.780)	0.405	1.715 (0.481–6.109)
	≥ 50,000 €			1		1
**Children (≤ 13 years old) in the household of respondent**	Yes		0.533	0.723 (0.260–2.008)	0.693	0.843 (0.361–1.968)
	No			1		1

Significant results (*p* < 0.05) are reported in bold

*Cl* Confidence interval, *OR* Odds ratio

^a^The word “Unimportant” combines the answers “not important” and “not very important” - The word “Important” combines the answers “important” and “very important”

## Discussion

Vaccination is considered the most successful health measure both in human medicine and in veterinary practice. International guidelines of the vaccination of cats and vaccination experts (WSAVA, AAFP, ABCD) recommend that, whenever possible, all cats receive the benefit of vaccination. This not only protects the single subject, but also provides an indispensable ‘herd immunity’ that minimizes the likelihood of infectious disease outbreaks [1, 2, 4].

This study aimed to understand factors influencing the Italian cat owners’ opinion about vaccination.

### Sample representativeness linked to the methodology

First of all, in our work we used a web-based questionnaire, as in other similar studies [7, 16]. Indeed, the web-based questionnaire allowed us to include both vaccinated and unvaccinated cats because it was spread online and not among veterinarian practices. Moreover, considering that the majority (65%) of the Italian population uses internet [17], online survey and social networks approach is a cheap and efficient way for obtaining data across a huge area in a short time: 84% of respondents found out about the questionnaire through the web.

However, considering the nature of internet-based surveys and advertisements conducted via social media networks, there is the possibility that the sample acquired in this study was not truly representative of the general pet owner population and thus our estimates may be biased.

Indeed, it is possible that owners searching information about pets on social media may be more “health conscious” and more prone to vaccination, even if information on health status of cats is obtained online by the majority (68%) of pet owners, regardless of compliance with vaccination of their cats [18]. Therefore, people not interested in pet health would not self-enroll in such a survey. Even though it was clearly written at the beginning of the questionnaire that the survey was aimed at all cat owners, also (and above all) at those who did not vaccinate their animals, it is possible that mainly owners who normally vaccinate cats participated in the study. This could explain the particularly high percentage of vaccinated cats found in our study. Results of phone-based or face-to-face surveys tend to justify this aspect with lower levels of pet health monitoring [19, 20].

Furthermore, the sample of cat owners responding to the survey was based on self-selection and this may have also introduced the age and gender bias observed in our study.

The young age bias probably reflects the common use of the internet by younger owners compared to the elderly. The strong bias towards female pet owners was not reported in previous studies [7, 16], even if females are reportedly more likely to keep pets than males [16, 21–23]. Females have been reported to be more incline to answer questionnaires and to use social networks than males in Italy [24] and this may have accounted for the high prevalence of female respondents (92%) observed in this study. Indeed, our gender bias was in line with a recent study based on a questionnaire disseminated online to English-speaking pet owners regarding pet nutrition [21]. Moreover, females have been reported to have higher empathy towards animals, be more interested in health-related topics and carry the primary responsibility for pets’ healthcare compared to males, and this may have increased the proportion of vaccinated cats compared to previous studies [16, 24].

Finally, questions to confirm recipients understanding of the word “vaccination” were not asked in our study and whether the participants in the survey completely understood what vaccination was and if they could differentiate it from other procedures performed by veterinarians is not known. It is possible that this may represent another limitation of this study [16].

Finally, our questionnaire was based on owner’s response and was not focused on the households owning the cats. Indeed, management practices of the pets are probably determined at the household level rather than by the single respondents. Comparing demographic characteristics of the sample and available national statistics for the household composition, household income, socio-professional category and living area show us that in some respects our population may not represent general population, and this could change the vaccination status of cats in Italy. For example, in our study respondents older than 50 years old represent only 23% while they represent 45% of the Italian population; Also, the population from north Italy corresponds to 38% of the national data and in our study, they represent 73% of the sample. This could be also correlated with the annual household income that varies a lot from north and south in Italy, being the medium income around 35,000 euros in north regions and 25,000 in south ones [25]. In fact, in this study more than 45% of the respondents indicated an annual income higher than 30,000 euros, and almost 60% of those have an annual income higher than 40,000 euros, clearly representing the impact of the overrepresentation of respondents form north Italy regions that are richer than the south ones.

Therefore, our results should be interpreted taking into consideration all these possible limitations of the study.

### Results obtained through the sample

The very high percentage (86%) of the owners that had taken their cats for vaccination to a veterinarian at least once showed Italian owners trust in cats’ vaccination. This result was higher than previously reported in other similar studies [19, 20], but the differences can be explained by both the considered study area and the different target populations. Indeed, owners living in semirural areas of central Italy and owners of cats living mainly outdoor have been reported to be less careful to cat’s health and this may account for the low percentages of cats that had veterinary consultation in these studies [19, 20].

Italian market studies concerning the pet-owner relationship provide conflicting results in terms of pet vaccination. In a study of 2017 [26] conducted on a sample of 1000 owners, 80% of them find it important that their pets are well cared, but only 46% vaccinate them regularly and 42% take pets to the vet only if they are ill. Data emerging from another market study in 2019 [5] show a completely different output: nearly 90% of the cat owners of the sample analyzed declares to regularly carry out feline vaccinations suggested by their veterinarian. Finally, the results of another survey conducted in the same year [27] showed that only 30% of cat owners go to the veterinarian once a year for pets’ check-up and vaccination.

The high percentage (80%) of owners that had their cat recently vaccinated within a three-year interval was in line with the 78% of recently vaccinated cats in Germany [7]. Our results were not comparable with the 69% of vaccinated cats in the UK because the interval was set in the preceding 12 months in the English study [16]. The three-year interval was used in this study to differentiate the recently vaccinated and the not recently vaccinated groups of cats. It must be reminded that the general three-year interval recommended by the current guidelines is applicable only to core vaccinations and needs to be considered depending on the vaccine and cats’ lifestyle, as previously observed [1, 3, 4, 7, 28–30].

Due to the high percentage of recently vaccinated cats and the low percentage of not adequately vaccinated animals (not recently vaccinated and unvaccinated cats), our results suggest that the cat population of this study may be well protected (considering the aforementioned “herd immunity” concept). However, it has to be reminded that a recent vaccination may not necessarily imply that the cat is well protected [28, 31]. Our results also suggest that the non-vaccination movement that has been reported in Italy in human medicine doesn’t seem to have, at least for now, a big impact in veterinary medicine and among pet owners in which trust in the veterinarian prevails.

Regarding the results from the multinomial logistic regression, the likelihood of recent vaccination being higher in cats between 2 and 4 years was surprising but was in accordance with a previous study [7].

Our results also showed that young cats (8 weeks-1 year of age) tended to be associated with the unvaccinated status. Therefore, it is of paramount importance to clarify to owners the necessity of vaccination in kittens starting at the age of 6–8 weeks, following the recommendations for a strong immunity.

Our results showing that older cats are more likely grouped within the not recently vaccinated group are in agreement with previous findings [7]. This result may be explained by owners thinking that older cats do not need to be vaccinated, because of a long-lived immunity following vaccination and a higher protection against infectious diseases. However, since old cats are known to have the same risk of infectious disease as younger cats, and ageing is associated with a decline in functional competence of the immune system, regular boosters are recommended for cats, regardless of their age [1, 2, 4, 28, 32].

Our analysis also showed that the annual household income had a significant impact on the vaccination status. Indeed, the likelihood of a recent vaccination status was significantly higher in cats which owners had a higher annual household income, whereas not recently vaccinated cats and unvaccinated cats were significantly associated to a lower household income. The annual household income has not been reported previously as a factor affecting the vaccination status of cats in the UK and in Germany [7, 16]. The difference with foreign countries may be explained by the fact that Italian owners are accustomed to pay for all veterinary services whereas 40% of cats have health insurance in the UK and the insurance covers most of veterinary services [16]. Moreover, the median household income in Italy is lower compared to that of Germany and the UK [33], and payment of veterinary services, including vaccinations, may be troublesome, especially for owners with lower household income. The economic factor may have also accounted for the significantly higher likelihood of cats being unvaccinated among owners who perceived vaccination cost as an important factor. The importance of vaccination cost has been previously reported to be associated with the unvaccinated status of cats in the UK [16]. Economic data should be carefully considered in the future due to the economic loss linked to the COVID-19 pandemic that may also reduce the likelihood of cats’ vaccination.

Concerning the likelihood of recent vaccination status, the significantly higher recent vaccination status among cats from breeders or pet shops compared to other cats was not surprising. High purchase price could lead owners to be more prompt to vaccination. Our results did not confirm the likelihood of a vaccination status higher in cats that had traveled abroad, visited a cat show or a cattery, probably due to the low number of cats belonging to these categories. These factors have been associated with the requirement of up-to-date vaccinations and European Pet Passport with vaccination against rabies to travel within Europe and are usually more common in purebred cats [7, 16]. The lower importance of cat’s stress and the higher perception of vaccination effectiveness reported by owners of recently vaccinated cats, even if not statistically significant, may also explain their willingness to vaccinate their cats.

The likelihood of not recent vaccination status was significantly higher in owners that perceived the lifestyle of cats as an important factor. This result is likely linked to the higher frequency of indoor lifestyle only in cats belonging to the not recently vaccinated group. This result suggests that owners of these cats might have assumed that animals living for prolonged periods in closed environments with no contact with other cats were not at risk and did not need revaccination. However, global guidelines recommend vaccination booster every 3 years also for cats living indoors only, except for FPV that may be administered every 3 years or more [1, 2, 4, 28, 29]. Veterinarians should consider this result and, to improve the vaccination status of cats, educate owners on the importance of adequate vaccination protocols and boosters also for cats living indoors only.

The likelihood of vaccinated status was higher in owners with health related jobs, such as doctors, nurses, or pharmacists, and that was not surprising. This result may also be linked to the lower level of education that was observed in the owners of the unvaccinated cats. Our results suggest that jobs not related to health aspects and lower education levels may be associated with little knowledge on scientific aspects, including the importance of vaccination, and therefore the higher unvaccinated status of cats is more likely.

The unvaccinated status tended to be associated with owners being 21–49 years old. Despite this was not a significant result, it is interesting to keep on monitoring this age category of owners because non-vaccination movements in Italy tended to be more frequently reported among people aged 25–44 [34].

The importance of factors preventing owners from having their cats vaccinated, such as cats’ capture, travel to the veterinarian and inappropriate waiting times, may be related to the lower perception of infectious risk and knowledge of importance of vaccination. Therefore, it is likely that owners of unvaccinated cats consider taking the cat to the veterinarian not necessary for their cat’s health. Indeed, such factors have been considered not important by a high percentage of owners of recently vaccinated cats. Moreover, the lower compliance of owners of unvaccinated cats with veterinary advice for vaccination and the lower importance for cats’ susceptibility to infectious disease compared to the owners of vaccinated cats, even if not statistically significant, may also explain why owners of unvaccinated cats do not understand the need of vaccination in cats. It should also be taken into consideration that both “unvaccinated” and “not recently vaccinated” categories could be at risk for infectious diseases, since vaccination is not up-to-date.

This study showed that Italian cats’ owners have a good level of confidence in veterinarians. Most owners reported the importance of veterinary advice and considered veterinarians as the main source of information for their cats’ health. More than half of respondents (59%) followed their veterinarian recommendation for annual vaccinations, which was surprising, as not in line with guidelines. Annual vaccination is recommended for some of the non-core vaccines, such as feline leukemia virus (FeLV) only in high-risk cats, *Chlamydophila (Chlamydia) felis*, feline immunodeficiency virus (FIV) and *B. bronchiseptica* (the last two vaccines are not available in Italy) and might also apply for feline herpesvirus (FHV-1) and feline calicivirus (FCV) in some high-risk situations [1, 2, 4, 28]. However, most cats of this study lived indoors (63%) and several lived in a single-cat household (31%), situations in which annual booster is not necessary. The only exception is for cats travelling abroad that, depending on the vaccine used, may require annual boosters of vaccination against rabies. Recommendation of annual vaccination was recently reported in German cats [7] and was suggested to be associated with lack of knowledge of feline vaccination guidelines or due to the old habit of veterinarians to vaccinate annually regardless of the type of vaccine used.

Results on the knowledge of antibody test to evaluate vaccination status and avoid unnecessary boosters showed that respondents who had vaccinated their cats were more aware of it and owners of recently vaccinated cats were more inclined to use it in the future.

## Conclusions

The first positive and important result of this study is the high number of recently vaccinated cats, that excludes, at least now, the existence of the risk of non-vaccination movements among cats’ owners. However, our sample may not be representative of the general population. Further investigation should focus on a detailed analysis of each cat’s vaccination history and a validation of the provided data that were not possible due to the high number of respondents and the anonymous nature of the questionnaire.

Our findings also suggest a growing attention towards feline vaccination and cats’ health as well as the routinely visit to the veterinarian and the possibility of a preventive antibody test to control the protection status instead of a blind vaccination. These results could be very important for veterinarians. First, the importance of veterinarians in client education has been confirmed, as owners trust veterinarians and rely on their recommendations. Moreover, knowing the difficulties of the owners, veterinarians could improve owners’ compliance (e.g., take appointment to reduce waiting time). Finally, keeping up to date with the vaccination guidelines will allow veterinarians to grow owners’ confidence and avoid useless revaccinations reducing costs that have been identified as important factors for owners.

## Methods

### Data collection

The online questionnaire was developed exclusively for this study and has not been previously published or used, and it included 31 questions about the following items: sociodemographic information about the respondents, vaccination history of the cats, factors influencing owners’ decision related to vaccination, experience of vaccination side effects in cats, relationship between cat owners and veterinarian and source of information about cat vaccination.

Owners were asked to rate factors that affected their decision in vaccination of their cat using a 4-points Likert scale for importance (not important, not very important, important, very important) and a 3-points Likert scale for usefulness (useless, useful, very useful). For the statistical analysis, the answers “not important” and “not very important” were grouped in the single word “unimportant”; in the same way the answers “important” and “very important” are grouped in the single word “important”. Always for the statistical analysis, the answers “useful” and “very useful” were grouped in the single word “useful”.

Few (*n* = 6) questions included an open-text option where owners could input their response. These included location of residency and cats’ origin, vaccination side effects, factors influencing vaccination choice, reason not to go to veterinarian and owners’ job if not present among the proposed answers. Owners had the option of omitting answers, which resulted in some incomplete data sets (see Results).

The questionnaire was initially piloted on 10 volunteers and questions were refined as required to improve the clarity and relevance of the questions. The questionnaire was prepared through the online program Google Forms and the link was mainly publicized using social networks (e.g., Facebook, Instagram, Twitter, cat forums), but also sent by email to acquaintances and promoted through leaflets. Concerning social networks, the questionnaire was publicized especially on cat-concerning pages.

Veterinarians were not included to minimize selection bias, as the aim of this study was to collect data from a sample representative of the general cat-owning population. The survey was available online from 28th June to 1st October 2018 and took about 10 min to be completed.

### Vaccination status

In this survey owners were asked if their cat had been vaccinated or not. Cats that had never been vaccinated were classified as “unvaccinated”. Owners were also asked if cats had been vaccinated within or before the previous 3 years, according to the vaccination frequency suggested by the global guidelines for the core vaccines [1, 2, 4]. Cats that had been vaccinated in 2015–2018 period were classified as “recently vaccinated” whereas cats that had been vaccinated before 2015 were classified as “not recently vaccinated”.

Vaccination status distinguishing between diseases was not performed as the recommended vaccinations in Italy, as worldwide, are the core vaccinations (against FPV, FHV-1 and FCV); moreover, at the time of our study there were only trivalent vaccines for these diseases in Italy, and no monovalent or bivalent products were available; the only monovalent vaccine for cats was against FeLV to be used in high-risk cats.

### Data analysis

In this study a multinomial logistic regression (MLR) was used with cat’s vaccination status as dependent variable and divided in three categories: “recently vaccinated”, “not recently vaccinated” and “unvaccinated”. Multinomial logistic regression model is an extension of binary logistic regression [35, 36], and it is effective when we have a polychotomous categorical dependent variable. In an MLR model, the estimates of parameters can be identified and compared to a baseline-category of the dependent variable [37].

Responses to open questions, if considered similar in nature, were categorized (e.g. “teacher” and “professor” were put together in the same category), and some categories were also combined to improve the fit of the model for statistical analysis (e.g. owner’s age 21–29/30–39/40–49, owner’s age 50–59/over 60 were combined as years of age, “cat from shelter”/“stray”, “useful”/“very useful”, “not important”/“not very important”, “important”/“very important” factors influencing cat vaccination).

Univariable analyses were used to test the variables for association with vaccination status, and were performed using a multinomial regression model with only one independent variable at a time. Variables with a value of *P* < 0.2 were considered for inclusion in a multivariable model. After, a model was run on the response as a function of the predictors to ensure that there were no multicollinearity issues; only variables with variance inflation factors (VIF) < 3 were included in the model.

Variables were retained in the model if they were shown to improve the fit of the model significantly by assessing the change of deviance. The final multivariable model was based on 1247 respondents who provided information on all variables included in the model. A Goodness-of-fit test was also carried out to assess the goodness of fit of the final multivariable model (Table 7). A statistical significance of the model fitting information indicates that the full model represents a significant improvement in fit over the null model, while non-significant test results for the goodness-of-fit test, are indicators that the model fits the data well [38, 39].

**Table 7:** Model Fitting Information and goodness-of-fit

Model	Model fitting criteria	Likelihood Ratio tests		
	-2 Log Likelihood	Chi-square	df	Sig.
Intercept only	1109.205			
Final	884.674	224.531	34	< 0.001
Goodness-of-fit				
	Chi-square	df	Sig.	
Pearson	1408.249	1610	0.995	
Deviance	861.918	1610	1.000	

Survey responses were analyzed, and quantitative and qualitative data were reported as frequency (n) and percentage (%).

The statistical program SPSS version 25.0 was used. Variables with *p*-value ≤0.05 were considered to be statistically significant, whereas tendency was considered in the presence of *p*-values > 0.05 but < 0.1.

## Supplementary Information

**Additional file 1.**

## Data Availability

The datasets used and/or analyzed during the current study are available from the corresponding author on reasonable request.

## Abbreviations

AAFP: American Association of Feline Practitioners
ABCD: Advisory Board on Cat Diseases
FCV: Feline Calicivirus
FeLV: Feline Leukaemia Virus
FHV-1: Feline Herpesvirus-1
FIP: Feline Infectious Peritonitis
FIV: Feline Immunodeficiency Virus
FPV: Feline Parvovirus
VGG: Vaccination Guidelines Group
WSAVA: World Small Animal Veterinary Association
