# Supplementary material for: A survey of Italian cat owners’ attitudes towards cat vaccination through a web-based questionnaire
Source: BMC Vet Res. 2021 Aug 9;17:267. doi: 10.1186/s12917-021-02981-z (PMC8350260; doi:10.1186/s12917-021-02981-z)
Supplement: Supplementary file 1 — Additional file 1. [file 12917_2021_2981_MOESM1_ESM.pdf]

Do you vaccinate your cat?

Welcome and thank you for having accepted to participate in this survey! The questionnaire is addressed at cat owners with the aim of evaluating their opinion on feline vaccination and then understanding how to spread better practices. By answering these short questions, in a completely anonymous form, you will allow us to also have your opinion. Participant data will remain anonymous and will only be used for didactics, research and statistical purposes. Thanks for your collaboration!

- How many cats do you own?
  - 1
  - 2
  - 3
  - ≥ 4
- How old is your cat?
  - 8 weeks-1 year old
  - 2-4 years old
  - 5-9 years old
  - ≥ 10 years old
- How old was your cat at acquisition?
  - Less than 8 weeks old
  - 8 weeks-1 year old
  - 2-4 years old
  - ≥ 5 years old
- Where was your cat from?
  - Animal shelter
  - Internet (social network, forum, blog, e-commerce web sites)
  - Relatives/friends/neighbours
  - Stray
  - Breeder
  - Pet shop
  - Farm
  - Other
- Is your cat a purebred cat?
  - Yes
  - No
- Where does your cat live?
  - Indoor only
  - Indoor with an outdoor access
  - Outdoor only
- Have you ever brought your cat (more than one answer is possible):
  - To visit a cattery
  - On a trip
  - To visit a cat show
  - None of the above
- Are you going to bring your cat (more than one answer is possible):
  - To visit a cattery
  - On a trip
  - To visit a cat show
  - None of the above

- Has your cat ever been vaccinated with one of these vaccines: Nobivac (Tricat Trio, Forcat), Purevax (RCP, RCP FeLV, RCPChFeLV), Versifel CVR, Fevaxyn Pentofel, Feligen CRP, Leucofeligen FeLV/RCP?
    - Yes
    - No

I VACCINATE MY CAT

- When was the last time you vaccinate your cat?
    - Within the past three years (2015-2018)
    - More than three years ago (before 2015)
- Why do you vaccinate your cat (indicate the main reason)
    - Vaccination is important for the health of my cat
    - Veterinary consultation
    - To receive certificate of vaccination
    - Suggestion from relatives/friends/neighbours
    - I read vaccination is important on internet/from mass media
    - I fear for my health and/or for the health of my family
    - Other
- The fact that you vaccinate your cat could depend on these factors: how important are they for you (4-point scale: not important, not very important, important, very important)?
    - Cost
    - Side effects
    - Stress of the cat
    - Veterinary consultation
    - Susceptibility of feline infectious diseases
    - Severity of feline infectious diseases
    - Efficacy of vaccination
    - Time involved for a vaccination
    - Lifestyle of the cat (indoor/outdoor)
    - Age of the cat
    - Disease or therapy of the cat
- Which one of these vaccine side effects did you notice (more than one answer is possible)?
    - Lethargy
    - Inappetence
    - Injection site reaction
    - Fever
    - Vomiting
    - Diarrhea
    - Lameness
    - None of the above
    - Other
- How severe were the side effects?
    - Lethargy
        - Insignificant and rare
        - Insignificant and common
        - Significant and rare
        - Significant and common
    - Inappetence
        - Insignificant and rare
        - Insignificant and common

        - Significant and rare
        - Significant and common
    - Injection site reaction
        - Insignificant and rare
        - Insignificant and common
        - Significant and rare
        - Significant and common
    - Fever
        - Insignificant and rare
        - Insignificant and common
        - Significant and rare
        - Significant and common
    - Vomiting
        - Insignificant and rare
        - Insignificant and common
        - Significant and rare
        - Significant and common
    - Diarrhoea
        - Insignificant and rare
        - Insignificant and common
        - Significant and rare
        - Significant and common
    - Lameness
        - Insignificant and rare
        - Insignificant and common
        - Significant and rare
        - Significant and common
    - Other
        - Insignificant and rare
        - Insignificant and common
        - Significant and rare
        - Significant and common

I DO NOT VACCINATE MY CAT

- Why do not you vaccinate your cat (indicate the main reason)?
    - Cost
    - I fear for side reactions
    - Vaccination is not important for the health of my cat
    - Vaccination is harmful for the health of my cat
    - Vaccination is not effective
    - Vaccination is stressful for my cat
    - Age of the cat
    - Lifestyle of the cat (indoor/outdoor)
    - Time involved for a vaccination
    - Disease or therapy of the cat
    - I did not know cats should be vaccinated
    - I do not care about vaccination
    - Oher

- The fact that you do not vaccinate your cat could depend on these factors: how important are they for you (4-point scale: not important, not very important, important, very important)?
  - Cost
  - Side effects
  - Stress of the cat
  - Veterinary consultation
  - Susceptibility of feline infectious diseases
  - Severity of feline infectious diseases
  - Efficacy of vaccination
  - Time involved for a vaccination
  - Lifestyle of the cat (indoor/outdoor)
  - Age of the cat
  - Disease or therapy of the cat

VACCINATION

- How often does your veterinarian recommend you vaccinate your cat?
  - Annually
  - Every two years
  - Every three years
  - More than three years
  - Unknown
  - My veterinarian has never mentioned it
- Which is the main deterrent from making a veterinary appointment?
  - Opening hours
  - Time involved
  - Distance to clinic and transport
  - Finding and catching the cat
  - None of the above, I usually make veterinary appointment
  - Others
- How important are these sources of information about feline vaccination (3-point scale: unhelpful, helpful, very helpful)?
  - Internet
  - Books
  - Relatives/friends/neighbours
  - Breeder
  - Veterinarian
  - Pet shop
  - Animal organization
  - Your own doctor
  - Pharmacist
- Antibodies tests can assess the level of protection of the cat after vaccination. Do you know this type of exam?
  - Yes
  - No
- Would you be willing to use it and vaccinate your cat just in case of not adequate protection?
  - Yes
  - No

THE OWNER

- How old are you?
  - ≤ 16 years old
  - 17-20 years old
  - 21-29 years old
  - 30-39 years old
  - 40-49 years old
  - 50-59 years old
  - ≥ 60 years old
- What is your gender?
  - Female
  - Male
- Where do you live?
  - City (≥ 15.000 residents)
  - Town (≤ 15.000 residents)
- Which is the postal code of your city?
- Which is your level of education?
  - Primary school certificate
  - Middle school certificate
  - High school certificate
  - Bachelor’s degree
  - Master’s degree
  - Post-university degree
- What do you do?
  - Breeder (dogs/cats)
  - Veterinarian
  - Doctor
  - Student in Veterinary Medicine
  - Student in Medicine
  - Other
- Which is the highest level of education in your family?
  - Primary school certificate
  - Middle school certificate
  - High school certificate
  - Bachelor’s degree
  - Master’s degree
  - Post-university degree
- Which is the annual household income of your family?
  - ≤ 9.000 €
  - 10-19.000 €
  - 20-29.000 €
  - 30-39.000 €
  - 40-49.000 €
  - ≥ 50.000 €
- How many children (≤ 13 years old) are in your family?
  - None
  - One
  - Two
  - Three
  - More than three

- How did you know this questionnaire?
    - Internet
    - Veterinarian
    - Relatives/friends/neighbours
    - Other
